# Changes in the expression of aromatase, estrogen receptor α and β in mandibular condylar cartilage of rats induced by disordered occlusion

**DOI:** 10.1186/1471-2474-13-190

**Published:** 2012-09-30

**Authors:** Shibin Yu, Xianghui Xing, Kai Jiao, Lei Sun, Lei Liu, Meiqing Wang

**Affiliations:** 1 School of Stomatology, Fourth Military Medical University, Xi’an, China

## Abstract

**Background:**

Estrogens play an important role in modulating the morphology and function of temporomandibular joints (TMJs), which is suggested to act via estrogen receptors (ERs). The present study was to investigate the expression of aggrecan, collagen type II (Col II), Col X, aromatase, ERα and ERβ in degenerative changes of mandibular condylar cartilage.

**Methods:**

Forty male and 40 female 8-week-old rats were enrolled in this study. In experimental groups, the disordered occlusion was created by moving the first molars mesially and the third ones distally. Immunohistochemistry and real-time PCR were performed at the end of the second or fourth week.

**Results:**

Degenerative changes, characterized by interrupted continuity of hypertrophic layer, pyknotic and eosinophilic lesion with few nuclei, areas filled with eosinophilic nuclei, were observed in more joints from female experimental groups than male ones. However, thickening changes in hypertrophic layer were only found in male experimental groups. The gene expression of Col II, Col X and aggrecan increased in 4-wk male experimental subgroup (both P < 0.01), but decreased in 2-wk and 4-wk female subgroups (P < 0.05). The gene expression of ERα decreased in 2-wk male and female experimental subgroups (both P < 0.01), however, that of ERβ increased except the 2-wk female experimental subgroup (all P < 0.01). The expression of aromatase decreased in both male and female experimental subgroups (all P<0.01).

**Conclusions:**

Mandibular condylar cartilage responses differently to the disordered occlusion in male and female rats. The levels of locally synthesized estrogen, ERα and ERβ may have limited attribution, if any, to the sex-specific cartilage response.

## Background

Temporomandibular disorders (TMD) is a collective term that embraces a number of clinical conditions that involve the masticatory musculature, temporomandibular joint (TMJ) and associated structures [1]. Many epidemiological studies have shown that the predilection of TMD in women is striking. The prevalence of TMD is 2 to 5 times higher in women than in men in community samples. The differential prevalence is even greater in patient populations [1-8]. A large proportion of women with TMD are between 18 and 45 years of age, different from the similar diseases of other joints that have a greater female predilection but occur postmenopausally [6]. In the past several decades, the role of female reproductive hormones in TMJ, especially estrogen, has attracted many researchers because of this marked sexual dimorphism and age distribution. In TMJs from ovariectomized animals the increased condylar cartilage thickness and even degenerative changes are noticed [9-12]. Studies on the effect of different concentrations of exogenous estrogen on cultured TMJ cartilage blocks or chondrocytes have also suggested that estrogen plays an important role in modulating the morphology and function of TMJs [13-15].

In literature, various studies have shown that estrogens synthesized by aromatase-expressing bone cells may have important effects at the skeletal tissue level, independent of the circulating levels of estrogens [16,17]. Our previous studies also showed that the level of local estrogen in condylar cartilage is less associated, if there is, with the serum estrogen [17,18]. A possible mechanism has been suggested that estrogen affects articular cartilage metabolism directly via estrogen receptors (ERs) in chondrocytes [19]. ER is one member of a family of activated transcription factors that can initiate or enhance the transcription of genes containing specific estrogen response elements [20]. It has been reported that there are two subtypes of ERs, ERα and ERβ, which are distinct proteins encoded by separate genes located on different chromosomes. Our previous study has identified that ERα and ERβ co-existed in rat mandibular condylar cartilage [18].

TMJ is a load-bearing structure with its load mainly from elevators, the function of which is adjusted by occlusion through feedback from mechanoreceptors in periodontal tissues. The articular surface of the TMJ is covered by fibro-cartilage that is distinct from the hyaline cartilages of other joints [21]. It is assumed to have a remarkable remodeling capacity that may be necessary in the functional adaptation for the whole life to mandibular growth or changes in dentition [22-24]. It has been proved by previous studies that structural remodeling changes of TMJ were associated with occlusion [25-27]. Unfortunately, the molecular events that underlie TMJ remodeling and adaptation, and whether ERs are involved in these procedures are poorly understood. In recent years, significant degenerative changes were observed in rat mandibular condylar cartilage induced by specially designed disordered occlusion [28-30]. The hypothesis of the present study is that there was close relationship between the expression of main extracellular matrix --aggrecan and collagen type II (Col II) and the expression of locally synthesized estrogen and ERs in the degenerative changes of mandibular condylar cartilage.

## Methods

### Animals and occlusal treatment

The study was approved by the Animal Research Committee of the Fourth Military Medical University. Forty male and 40 female 8-week-old SD rats, weighing 200-220g in male and 180-200g in female, were provided by the animal center of the Fourth Military Medical University. All animals were randomly divided into experimental group and control group, twenty for each group.

All animals were housed under conditions of 22°C and 30-60% relative humidity with a normal day-night rhythm, consisting of a 12:12 hour light–dark cycle. The rats had free access to tap water and standard food, and no dietary adjustments were made. Before the beginning of the experiment, all the animals were bred for 2 days to adapt to the environment.

In experimental group, occlusal treatment, which has been proved to induce obvious degenerative changes of mandibular condylar cartilage successfully, was carried out with a method as previously reported [28,30]. Briefly, Under anesthesia with pentobarbital sodium injected intraperitoneally (25mg/kg body weight, Sinopharm Chemical Reagent Co., Ltd, China), an elastic rubber band (Unitek^TM^ Elastics, 3M Unitek, 1/8#), about 1 mm in diameter, was inserted between the first and second molars of the left side of maxillary dentition and the right side of mandibular dentition. The rubber bands were carefully placed at the level lower than the occlusal surface of the molars so that occlusal contact relationship would not be interfered. The first molars were gradually moved mesially, with a relatively stable gap about 0.8 mm wide 7 days later. Then, to keep this gap till the end of the experiment, the elastic bands were replaced by self-curing resin (Zhangjiang Biomaterial Co., Shanghai, China). Care was taken to prevent the resin to contact with opposing molars. At the beginning of the 5^th^ week, the same method was used to move the left maxillary and right mandibular third molars distally. In all the occlusal treatments, the length of mouth opening was less than 15mm, with the time of each operation less than 5 minutes. In control group, all the rats were subjected to all the procedures, except for inserting the rubber bands or placing self-curing resin between the molars. Animals in experimental group and their age-matched controls were sacrificed at the end of the second or fourth week after the inserting the last rubber band, named as the 2-wk and 4-wk subgroups. During our observed experimental period, there was no significant difference in feed intake or body weight between experimental and sham groups.

### Tissue preparation

Under deep anesthesia with intraperitoneal injection of pentobarbital sodium (50 mg/kg body weight), for morphological analysis and immunohistochemistry, four rats in each group were perfused with 200ml normal saline through ascending aorta, followed by 400 ml paraformaldehyde (4% in phosphate buffer saline, pH 7.4). The TMJs were dissected and post-fixed overnight at 4°C with the same fixative, and then decalcified for 1 week in Kristensen’s fluid (sodium formate 52.2g, formic acid 174.2ml, 1000ml distilled water). TMJ samples were then dehydrated in graded ethanol and then embedded in paraffin. Serial mid-sagittal sections of 5 μm in thickness were cut parallel to the lateral surface of the condyle neck of the mandible ramus. For histological observation, one section from each joint was stained with hematoxylin and eosin. For the real-time PCR analysis, the other six rats in each group were sacrificed. To get enough total RNA, four condylar cartilages from 2 randomly selected rats were considered as one sample, so 12 condylar cartilages from 6 rats in each group were randomly assigned into 3 samples.

### The area measurement of degenerative regions

All measurements were made by one examiner (Lei Liu) who knew nothing about the grouping of animals. The sections stained with HE were observed and photographed under microscope (Leica DM 2500). A true-colour computer-assisted image analyzing system with a digital camera (Leica DFC490, Leica, Wetzlar, Germany) and software (Qwin Plus, Leica Microsystem Imaging Solusions Ltd, Cambridge, United Kingdom) were applied for images capture and measurement. As we previously reported [29], the total areas of the condylar cartilage and the degenerative region in the cartilage were quantified by outlining the periphery of the entire cartilage and the degenerative region in the cartilage. Area measurements were made three times, and the averaged data were used to calculate the percentages of OA-like degenerative regions. The percentages were then ranked for statistical analysis as follows: 0, 0%; 1, 0%-10%; 2, 10%-20%; and 3, more than 20%.

### Immunohistochemical staining

Five commercially available primary antibodies were used in immunohistochemical staining. They were anti-human Col II goat polyclonal IgG (SC7763, Santa Cruz Bio. Inc., USA), anti-rat Col X rabbit polyclonal IgG (LSL-LB-0092, Cosmo Bio Co., Ltd, Japan), anti-human ERα rabbit polyclonal IgG (SC542, Santa Cruz Bio. Inc., USA), anti-rat ERβ rabbit polyclonal IgG (ab3577, Abcam, UK), and anti-human aromatase goat polyclonal IgG (SC14245, Santa Cruz Bio. Inc., USA). Four sections from the middle part of each joint were selected for this study. Immunohistochemical staining was carried out with a three-step avidin-biotin complex method as described previously [18]. Briefly, after deparaffinization and rehydration, the sections were treated with 3% hydrogen peroxide at room temperature for 10 min to eliminate endogenous peroxidase activity. Then the antigenic sites were exposed by digestion with Antigen Retrieval Solution (Wuhan Boster Biological Technology Ltd. China) for 10 min, and nonspecific binding sites were blocked by incubating the sections with normal serum for 30 min at 37°C. The sections were incubated overnight at 4°C with ①anti-human Col II goat polyclonal IgG (3 ug/ml), ②anti-human ERα rabbit polyclonal IgG (4μg/ml), ③anti-rat ERβ rabbit polyclonal IgG (6μg/ml), ④anti-human aromatase goat polyclonal IgG (4ug/ml). The bound primary antibody was then localized by biotin-labeled IgG (BeiJing ZhongShan Golden Bridge Biotechnology Co., Ltd., China) at 37°C for 30 min and then an avidin-peroxidase complex at 37°C for 30 min. The antibody staining was performed using peroxidase/diaminobenzidine (DAB) yellow kit (Wuhan Boster Biological Technology Ltd., China). The sections were lightly counterstained with hematoxylin, and then dehydrated in ethanol series, cleared in xylene and coverslipped. A control study of immunohistochemical staining was performed using the same staining procedure without the primary antibodies. No immunoreactivity was observed in these particular sections (data not shown).

### RNA preparation, reverse transcription, and real-time PCR

Mandibular condylar cartilage samples were pulverized in liquid nitrogen. The total RNA was isolated from frozen tissues using a standard TRIzol® protocol (Invitrogen, Carlsbad, CA), followed by first-strand cDNA synthesis with the RevertAid^TM^ First Strand cDNA Synthesis Kit (Fermentas). Real-time PCR was performed in ABI 7500 Fast thermal cycler. The protocol comprised 40 cycles of 94°C for 5 sec, 62°C for 34 sec, and 72°C for 1 min each. The detected cytokines were Col II, Col X, aggrecan, ERα, ERβ and aromatase. Table 1 shows the sequences of primers used in this study. Ribosomal protein S18, a housekeeping gene, was chosen as the reference gene in the present study. A ▽CT value was calculated for each sample by subtracting the threshold cycles (CT) of the S18 from the CT value of the detected gene. All samples in each group were normalized to the ▽CT value of a control sample (▽▽CT). The relative expression was calculated using the expression 2^-▽▽CT^ and reported as arbitrary units [31].

**Table 1:** Primer sequences for Col II, Col X, aggrecan, ERα, ERβ, aromatase and S18

**Gene**	**Sequence**	**Fragment length (bp)**	**Accession number**
Col II	F: 5’-AGAACTGGTGGAGCAGCAAGA-3'	124 bp	NM_012929
	R: 5’-ATCTGGACGTTAGCGGTGTTG-3’		
Col X	F: 5’- CCATGGTTCACACAACCCCTT -3’	129 bp	AJ131848.1
	R: 5’- TGGCTGTGGTAAAGCACCTTG -3’		
Aggrecan	F: 5’-CCCTCACCCCAAGAATCAAGT-3’	178 bp	NM_022190
	R: 5’- TCATTGGAGCGAAGGTTCTGG-3’		
ERα	F: 5’-TGCGCAAGTGTTACGAAGTGG-3’	108 bp	NM_012689
	R: 5’-TTCGGCCTTCCAAGTCATCTC-3’		
ERβ	F: 5’-AAAAACTCACCGTCGAGCCTT-3’	124 bp	NM_012754
	R: 5’-GCTGAATACTCATGGCGGTTG-3’		
Aromatase	F: 5’-TCATCAGCAAGTCCTCGAGCA-3’	106 bp	M33986
	R: 5’-CCATTCTCGTGCATGCCAAT-3’		
S18	F: 5’-CGGCTACCACATCCAAGGAA-3’	187 bp	M11188
	R: 5’-GCTGGAATTACCGCGGCT-3’		

### Statistical analysis

The results were quantified or evaluated by one examiner (Lei Liu) who knew nothing about the grouping of animals. The SPSS 11.0 package (SPSS Inc., Chicago, IL, USA) was used to analyze and describe the data. Three-factor analysis of variance with full interaction was adopted. The fixed factors were treatment (control and experiment), time point (2-wk and 4-wk), and sex (male and female). When significant main effects or the interaction between main effects were found, specific comparisons between groups were made by Student’s t test. For the results of the ranked data about the percentages of OA-like degenerative regions, were compared by using the nonparametric Mann–Whitney U test. P-values were considered to be statistically significant when less than 0.05.

## Results

### The morphology of mandibular condylar cartilage

**Figure 1:**
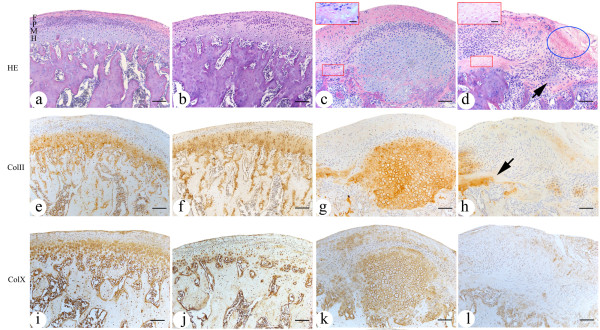
**The histologic morphology** (**a, b, c, d) and the expression of Col II (e, f, g, h), Col X(i, j, k, l) in the middle and posterior regions of mandibular condylar cartilage.** (**a**, **e**, **i**) and (**b**,** f**, **j**) were from 4-wk male and female control subgroups (16-week-old) separately. All layers of condylar cartilage aligned regularly, with good continuity in each layer. (**c**, **g**, **k**) and (**d**, **h**, **l**) were from 4-wk male and female experimental subgroups separately. As shown in (**c**), thickening changes, especially in hypertrophic layer, were observed. The continuity of hypertrophic layer was interrupted by the degraded area (in the red frame near subchondral bone), in which some nuclei became eosinophilic. As shown in (**d**), the layers of condylar cartilage were disarranged obviously, with the continuity of hypertrophic layer interrupted. The arrow showed one hypertrophic chondrocytes island surrounded by cells from other layers. The blue ellipse showed one degraded area characterized by pyknotic, homogeneous and eosinophilic lesion with few nuclei. Near subchondral bone, there was another degraded area full of eosinophilic nuclei (in the red frame). Immunoreactivity of Col II and Col X was observed mainly in hypertrophic and mature layers of condylar cartilage. As the arrow in (**h**) showed, no or weak immunoreactivity was found in degraded or disarranged areas. F=fibrous layer, P=proliferating layer, M=mature layer, H=hypertrophic layer. Scale bar is 100μm in Figure 1a–l, and 25μm in the enlarged red frames of Figure 1c and d.

In mandibular condylar cartilage of control group, fibrous layer, proliferating layer, mature layer and hypertrophic layer arranged regularly with good continuity in each layer (Figure 1a, 1b). However, obvious degenerative changes, characterized by interrupted continuity of hypertrophic layer, pyknotic, homogeneous and eosinophilic lesion with few nuclei, and areas filled with eosinophilic nuclei near subchondral bone (Figure 1c and 1d), were observed in 2 and 4 joints of 8 joints from the female 2-wk and 4-wk experimental subgroups, and in 0 and 2 joints of 8 joints from the male 2-wk and 4-wk experimental ones. Furthermore, as shown in Table 2, the percentage areas of OA-like degenerative regions in 4-wk female experimental groups were higher than other control and experimental groups (P<0.05). However, thickening changes in condylar cartilage, especially in hypertrophic layer, were found in 1 and 3 joints of 2-wk and 4-wk male experimental subgroups, but none in female ones (Figure 1c).

**Table 2:** Distribution of ranked data of the area of OA-like changes as a percentage of total cartilage

**Rank**	**Con 2w**		**Exp 2w**		**Con 4w**		**Exp 4w**	
	**Male**	**Female**	**Male**	**Female**	**Male**	**Female**	**Male**	**Female** *
0	8	8	8	6	8	8	6	4
1	0	0	0	0	0	0	1	1
2	0	0	0	1	0	0	1	0
3	0	0	0	1	0	0	0	3
Total	8	8	8	8	8	8	8	8

The percentages were ranked as follows: 0, 0%; 1, 0%-10%; 2, 10%-20%; 3, >20%.

* P<0.05 vs other groups.

### The expression of extracellular matrix in mandibular condylar cartilage

In mandibular condylar cartilage of control group, the Col II and Col X immunohistochemical signals distributed continuingly in mature and hypertrophic layers, especially in hypertrophic layer (Figure 1 e, 1f, 1i and 1j). However, in the degenerative condylar cartilage, the continuity of Col II and Col X immunoreactivity was interrupted by degraded or disarranged areas, in which no or weak immunoreactivity could be found (Figure 1g, 1h, 1k and 1l).

**Figure 2:**
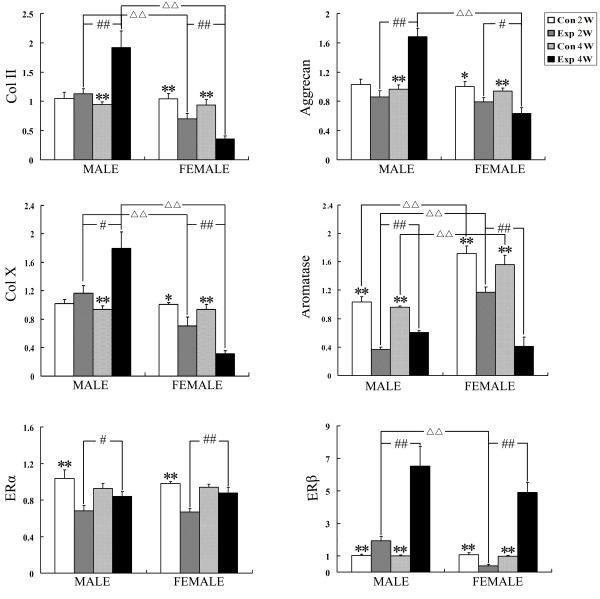
**Comparison of the gene expression of Col II, aggrecan, ERα, ERβ and aromatase in the mandibular condylar cartilage in different groups (n=3).** Error bars represent standard error. Levels of significance were determined using Student’s t-test. ^*^ P < 0.05 and ^**^ P < 0.01 indicate significant difference between experimental and age-matched control groups. ^#^ P < 0.05 and ^##^ P < 0.01 indicate significant difference between 2-wk and 4-wk experimental subgroups^. △△^ P < 0.01 indicate significant difference between male and their age and treatment-matched female subgroups.

Compared to their age-matched control subgroups, the gene expression of Col II, Col X and aggrecan increased significantly in 4-wk male experimental subgroups (all P<0.01), but decreased significantly in both 2-wk and 4-wk female experimental subgroups (P<0.05). The gene expression of Col II, Col X and aggrecan in 4-wk male experimental subgroups was higher than 2-wk male experimental subgroups (P<0.05), while that in 4-wk female experimental subgroups was lower than 2-wk female experimental subgroups (P<0.05). Additionally, the gene expression of Col II and Col X in 2-wk male experimental subgroups, and that of Col II, Col X and aggrecan in 4-wk male ones, was significantly higher than female age-matched experimental subgroups (all P<0.01) (Figure 2).

### The expression of ERα and ERβ in mandibular condylar cartilage

**Figure 3:**
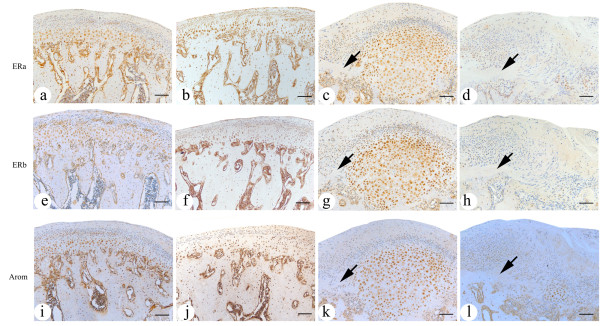
**The expression of ERα (a, b, c, d), ERβ (e, f, g, h) and aromatase (i, j, k, l) in the middle and posterior regions of mandibular condylar cartilage.** (**a**, **e**, **i**) The expression of ERα, ERβ and aromatase in condylar cartilage from 4-wk male control subgroup. (**b**, **f**,** j**) The expression of ERα, ERβ and aromatase in condylar cartilage from 4-wk female control subgroup. (**c**, **g**, **k**) The expression of ERα, ERβ and aromatase in condylar cartilage from 4-wk male experimental subgroup. (**d**, **h**, **l**) The expression of ERα, ERβ and aromatase in condylar cartilage from 4-wk female experimental subgroup. Immunoreactivity of ERα, ERβ and aromatase was observed in hypertrophic and mature layers of condylar cartilage. As arrows showed, no or weak immunoreactivity was found in degraded or disarranged areas. Scale bar is 100μm in Figure 1a- 1l.

In mandibular condylar cartilage of control group, the ERα and ERβ immunohistochemical signals distributed continuingly in mature and hypertrophic layers (Figure 3a, 3b, 3e and 3f). However, in the degenerative condylar cartilage, the continuity of ERα and ERβ immunoreactivity was interrupted by degraded or disarranged areas, in which no or weak immunoreactivity could be found (Figure 3c, 3d, 3g and 3h).

Compared to their age-matched control subgroups, the gene expression of ERα decreased significantly in 2-wk both male and female experimental subgroups (both P<0.01), but that of ERβ increased significantly in 2-wk male and 4-wk both male and female experimental subgroups (both P<0.01). However, the expression level of ERβ in 2-wk female experimental subgroup was significantly lower than its age-matched control group. Additionally, the gene expression of ERα and ERβ in 4-wk both male and female experimental subgroups was higher than 2-wk sex-matched experimental subgroups (P<0.05). Furthermore, the gene expression of ERβ in 2-wk male experimental subgroups, was significantly higher than female age-matched experimental subgroups (P<0.01). No such sex differences were found between other subgroups (Figure 2).

### The expression of aromatase in mandibular condylar cartilage

Similar to that of ERα and ERβ, the aromatase immunohistochemical signals distributed continuingly in mature and hypertrophic layers in mandibular condylar cartilage of control group (Figure 3i and 3j). However, in the degenerative condylar cartilage, the continuity of aromatase immunoreactivity was interrupted by degraded or disarranged areas, in which no or weak immunoreactivity could be found (Figure 3k and 3l).

As shown in Figure 2, the expression of aromatase in female control group was higher than their age-matched male one (P<0.01). Compared to their age-matched control subgroups, the expression of aromatase decreased significantly in both male and female experimental subgroups (all P<0.01). In male experimental group, the expression of aromatase in 2-wk subgroup was lower than 4-wk subgroup (P<0.01). However, the expression of aromatase in 2-wk subgroup was higher than 4-wk subgroup in female experimental group (P<0.01).

## Discussion

TMJ is loaded during mastication and other oral behaviors. The mechanical loads are vital for maintaining normal growth, morphology and function of TMJ cartilage [32-34]. It has also been reported by clinical studies that mandibular condylar cartilage remodels throughout the whole life to accommodate the changes in dentition, such as occlusal equilibration or orthodontics treatment [23,24]. It has been reported that degenerative changes of mandibular condylar cartilage can be induced by forced jaw opening in rats [35] (applied 1h per day for 20 days) and rabbits [36] (applied 3 h per day for 5 days). In the present study, the time of each operation was less than 5 minutes. More than that, significant degenerative changes were observed only in experimental groups, which implied that abnormal mechanical load was induced by the present disordered occlusion [28-30]. However, lack of the information about load translation and distribution on condyle, the present study can not clarify the relationship between actual load and the morphological changes of mandibular condylar cartilage. Investigation with methods like three-dimensional finite element analysis might provide valuable informations [37].

The extracellular matrix of the cartilage provides the unique biomechanical properties of articular cartilage. Progressive destruction of the extracellular matrix causes a failure of the cartilage [38,39]. The present study showed a lower gene expression of Col II, Col X and aggrecan in female experimental subgroups, with a further lower expression level in 4-wk subgroups than 2-wk ones. It indicates a progressive destruction of extracellular matrix. However, in male rats there was an increased gene expression of Col II, Col X and aggrecan in 4-wk subgroup. The increased gene expression may attribute to the thickening changes of condylar cartilage observed in male experimental subgroups. The sex difference in the extracellular matrix of the condylar cartilage, which is associated with loading suffering ability, is considered the explain, at least partially, for the female predominance in chondrocytes death as reported in our previous study [28] and the female predilection of TMD.

There is growing awareness that estrogen can be synthesized through estrogen synthetase - aromatase not only by gonadal glands but also by a number of extragonadal sites including mandibular condylar cartilage, in which estrogen can act locally in a paracrine and intracrine fashion [17,40]. Although the total amount of estrogen synthesized at any given site could be small, local concentrations, could be substantial, giving it functional meaning [40]. One study on clinical samples found that the expression of aromatase was reduced in the bone tissue of patients with severe OA, in comparison to patients with hip fractures [41]. In the present study, both OA-like degenerative changes and decreased expression of aromatase in mandibular condylar cartilage were induced by disordered occlusion in both male and female animals, which may indicate that lower levels of locally synthesized estrogen may play an important role in the degenerative changes of condylar cartilage. In literature, various studies suggest that estrogens may indeed influence the development of OA, and low levels of estrogens have been associated with an increased risk of OA in both experimental animals and humans [16]. Taken into consideration that thickening changes and even degenerative changes in condylar cartilage can be induced by ovariectomy [9-12], serum estrogen is also an attractive candidate in the degenerative changes in OA process.

It has been suggested that ERα and ERβ usually play different, even opposite, roles in bone tissues [42-44]. For instance, in periosteum (analogous to the condylar cartilage), ERα either increases bone formation in males or has no effect. In contrast, ERβ inhibits periosteal bone formation and moment of inertia in females but has no effect in males [42]. The facts that the expression of ERα decreased and the expression of ERβ increased in present experimental groups (except the 2-wk female subgroups) support the different roles of ERα and ERβ in the degenerative changes of mandibular condylar cartilage. Although the expression of extracellular matrix in male experimental subgroups (except aggrecan in 2-wk experimental subgroups) was significantly higher than their age-matched females, sex difference in the expression of ERβ was only found in 2-wk experimental subgroups, with no similar sex difference in the expression of ERs in other groups. It is suggested that the ERs have limited attribution, if any, to the sex difference in degenerative changes.

When interpreting the present results, one should keep in mind that the present OA-like lesions, as well as the expression of cytokines, cannot be equated with OA in human being because of the obvious differences in occlusion, as well as TMJ, between rats and human.

## Conclusions

In summary, in the process of degenerative changes within our observed experimental period, male and female rats response differently to the disordered occlusion, more degenerative changes and lower expression of extracellular matrix in female condylar cartilages, as well as more thickening changes and higher expression of extracellular matrix in male ones. The levels of locally synthesized estrogen, ERα and ERβ may have limited attribution, if any, to the sex-specific cartilage response. The detailed mechanisms in these processes need further investigation.

## Competing interests

The study was fully supported by the National Natural Science Foundation of China (No.30901699, No.30928028). The authors declare that they have no competing interests.

## Authors’ contributions

Meiqing Wang led the project and revised the manuscript. Shibin Yu carried out the histology, immunohistochemistry staining, and drafted the manuscript. Xianghui Xing carried out real-time PCR, and revised the manuscript. Kai Jiao performed the statistical analysis and helped to revise the the manuscript. Lei Sun carried out the animal model. Lei Liu carried out the animal model, and evaluated the results. All authors read and approved the final manuscript.

## Pre-publication history

The pre-publication history for this paper can be accessed here:

http://www.biomedcentral.com/1471-2474/13/190/prepub
